# A cluster randomized control field trial of the ABRACADABRA web-based reading technology: replication and extension of basic findings

**DOI:** 10.3389/fpsyg.2014.01413

**Published:** 2014-12-04

**Authors:** Noella A. Piquette, Robert S. Savage, Philip C. Abrami

**Affiliations:** ^1^Faculty of Education, University of LethbridgeLethbridge, AB, Canada; ^2^Department of Educational and Counselling Psychology, McGill UniversityMontreal, QC, Canada; ^3^Centre for the Study of Learning and Performance, Concordia UniversityMontreal, QC, Canada

**Keywords:** randomized controlled trial, reading development, web applications, internet, intervention, elementary school education

## Abstract

The present paper reports a cluster randomized control trial evaluation of teaching using ABRACADABRA (ABRA), an evidence-based and web-based literacy intervention (http://abralite.concordia.ca) with 107 kindergarten and 96 grade 1 children in 24 classes (12 intervention 12 control classes) from all 12 elementary schools in one school district in Canada. Children in the intervention condition received 10–12 h of whole class instruction using ABRA between pre- and post-test. Hierarchical linear modeling of post-test results showed significant gains in letter-sound knowledge for intervention classrooms over control classrooms. In addition, medium effect sizes were evident for three of five outcome measures favoring the intervention: letter-sound knowledge (*d*= +0.66), phonological blending (*d* = +0.52), and word reading (*d* = +0.52), over effect sizes for regular teaching. It is concluded that regular teaching with ABRA technology adds significantly to literacy in the early elementary years.

## INTRODUCTION

There is widespread deployment of information and communication technologies (ICTs) in schools around the world (Cuban, 2001; Chambers et al., 2008). Given this, how good is the evidence that technology actually can aid reading acquisition? Over the years there has been much research on this question. Several reviews of research exist that included experimental and quasi-experimental studies. These studies have generally identified small positive effect sizes for ICT use on literacy (e.g., Ehri et al., 2001; MacArthur et al., 2001; Blok et al., 2002; Cheung and Slavin, 2012). As a result, these authors are cautiously optimistic about the educational use of ICTs to produce small positive effects on literacy outcomes, particularly when technologies are deployed in close conjunction with teacher’s non-technology based efforts to improve literacy (e.g., Cheung and Slavin, 2012).

These overall findings are perhaps nuanced by a number of recent findings. More recently, Van Daal and Sandvik (2013) carried out a systematic review of all of the available literature and reported medium positive effect sizes for ICT use on literacy outcomes such as concepts of print and phonological awareness, suggesting these variables at an early stage of children’s literacy development are particularly amenable to technology-based intervention. Secondly, a recent tertiary meta-analysis of published meta-analyses of technology by Archer et al. (2014) suggests that under optimal conditions of extended teacher training and support (e.g., involving initial training and delayed re-training and with some initial just-in-time; classroom support), effect sizes for technology impacts on reading can be as high as +0.60, whereas under sub-optimal conditions of training such as with a single day professional development training session (or less) or where details of training are underspecified, the effect sizes are often closer to zero. Thirdly, it is crucial to bear in mind that the degree to which technology content reflects evidence-based practice will impact on literacy outcomes. Most of the popular programs evaluated to date for children beginning to read, for example, do not stand up well to such close scrutiny of their content validity (Grant et al., 2012).

In addition to these methodological issues one central issue about research design is highly relevant: in most areas of public policy there have been repeated calls for the use of best-quality evidence (e.g., Haynes et al., 2012). Understanding technology and its effectiveness is clearly a central issue for policy makers around the world (see e.g., all papers in this special issue). Archer et al. (2014) argued that high-quality randomized control trials (RCTs) must occupy a central role in understanding the effects of technology on literacy, as such methodologies are if well-executed, unique ways to ensure that the effects reported are due to the intervention rather than to extraneous factors. Some recent research studies using RCT designs have suggested that certain technologies can improve the reading skills of at-risk poor readers (e.g., Saine et al., 2011). Nevertheless, it is clear that more systematic evidence is needed on the issue of technology and its impacts on for literacy, and in particular for such interventions delivered to whole classes of children, using strong experimental and longitudinal methods, high quality measures, and interventions that are especially amenable to classroom use (e.g., Cheung and Slavin, 2012; Archer et al., 2014). Such systematic research moves program evaluation from researcher-delivered ‘efficacy’ trials (establishing internal validity) to teacher-delivered ‘effectiveness’ trials in ecologically valid contexts (thereby establishing external validity).

### THE ABRACADABRA WEB-BASED LITERACY PROGRAMMATIC RESEARCH

ABRACADABRA, an interactive tool, is designed as a support for teachers and parents to help young children develop fundamental early literacy skills. ABRACADABRA (hereafter, ABRA), is an acronym: *A Balanced Reading Approach for Canadians (now, Children) Designed to Achieve Best Results for All.* ABRA has been utilized within the early years of schooling, providing a user-friendly, free of charge, evidence-based tool to enhance literacy instruction and promote the development of children’s literacy, especially among struggling readers. ABRA was developed by a team of literacy and classroom technology experts through the Centre for the Study of Learning and Performance (CSLP) at Concordia University, Montreal (see Hipps et al., 2005; Abrami et al., 2008, 2010 for details). ABRA was originally developed by drawing upon the recommendations from the United States’ (National Reading Panel and National Institute of Child Health and Human Development, 2000) along with the Canadian Language and Literacy Research Network’s (2009) recommendations and has been refined and expanded annually. The ABRA program can be used flexibly as the activities are organized into the foundations of literacy acquisition, including alphabetic letter and sounds, fluency, comprehension, and writing categories. Furthermore, the complete version of ABRA includes student, teacher, assessment, and parent modules designed to target specific skills for instruction and guide students to progress from basic sound and letter identification to increasingly complex tasks such as spelling or narrative responses to stories.

In sum, ABRA promotes the teaching and learning of early language and literacy English skills, especially those at risk of school failure. It consists of 32 instructional activities and 17 interactive stories that combine to create 100s of challenging and engaging tasks for learners at differing complexity levels. An increasing number of student stories from around the world have been added and pronunciations are made in Canadian, Australian, and Kenyan English dialects. A French prototype of ABRA will be released publicly in short order and it’s instructional and assessment modules continue to be expanded ^1^. Finally, an electronic library of free digital stories called “Repository of Electronic and Digital Stories” (READS) has been created as a supplement to ABRA to help reinforce the development of fluency skills. This game-like interactive free access literacy tool can be downloaded ^2^ for home or school use or alternatively, downloaded and stored on a school board server ^3^ in order to also access the assessment and communication modules.

ABRA has been the subject of numerous validation studies including several ‘true’ (randomized control trial) experimental studies. First, in a within-classroom RCT design, 53 children from a disadvantaged English as a Second Language, low SES urban kindergarten setting, the students were systematically exposed to ABRA instruction in small groups for 10 h over 13 weeks (Comaskey et al., 2009). Results of this research found that phoneme-based and rime-based teaching led to growth in these two domains. Second, Savage et al. (2009) carried out a 13-weeks study with 144 Canadian first graders who experienced significant advantages in letter-sound knowledge, phonological blending, listening comprehension, and reading comprehension. The students received the ABRA intervention in small groups for an average of 13 h per child delivered by trained research assistants (RAs) while integrating ABRA into the regular reading classes. In the third within-class RCT evaluating ABRA, 300 students, including numerous aboriginal children in the Northern Territories in Australia with English as an Additional Language were taught by specially trained teachers who delivered ABRA as a pull-out program in schools for 40 min four times a week for 16 weeks (Wolgemuth et al., 2013). Results of this study found that the students who received the ABRA interventions showed significant advantages in phonological awareness and grapheme-to-phoneme knowledge. In a recent intervention study using ABRA over 13 weeks with grade 2 children in Kenya (Abrami et al., 2014), children were brought by bus to a technology center to use ABRA. Advantages were reported at post-test in children’s reading comprehension skills, suggesting that ABRA can be used in developing countries and also influence text-level comprehension as well as word-level alphabetic skills.

In a large Pan Canadian study, Savage et al. (2013) reported a Cluster RCT intervention study of the effectiveness of ABRA. This study used a classroom-level RCT intervention with 1067 children in 74 kindergarten and grade 1 or 1/2 classrooms across Canada, for 20 h per child over a school term while adhering to the CONSORT criteria for executing and reporting the highest quality RCT studies. Results demonstrate that the ABRA intervention classrooms were at a significant advantage over controls in standard measures of phonological blending ability, letter-sound knowledge, as well as marginally, for phoneme segmentation fluency. Additional analyses showed that with high fidelity of implementation (80% of intervention teachers), advantages were evident at post-tests in phonological blending, phoneme segmentation fluency, sight word reading, and letter-sound knowledge. This research suggests that ABRA is an effective resource for key skills associated with early language and literacy attainment. Other intervention studies have found that ABRA can moderate the associations between literacy and attention and may support students at risk for reading and attention difficulties (Deault et al., 2009), and that the ABRA exposed analytic phonics group performed better on a passage reading comprehension task than the synthetic phonics group (Di Stasio et al., 2012).

Most of the scientific evidence regarding ABRA described above came from trials run by university-based researchers and delivered by specially trained professionals rather than regular teachers in their typical classrooms as part of typical language arts classes. As such these former studies assess the internal validity or ‘efficacy’ of a tool under somewhat atypical and perhaps somewhat more optimal circumstances than usually obtain in schools. ABRA was designed as a tool for regular classrooms, so there is a need for studies under more natural conditions run by teachers in their classrooms as one part of a program of language arts. Methodologically, such field studies test the external validity or ‘effectiveness’ of tools. The only published ABRA study to date operating under these circumstances is the Savage et al. (2013) study, hence, there is a pressing need for replication and extension of these findings to confirm the external validity of ABRA. Thus, we explored this issue here.

The positive effects on early reading skills demonstrated in the ABRA research studies worldwide first drew the attention of a geographically remote northern Alberta school district to the current researchers. From their perspective, the school district was interested in training their teachers to run ABRA with direct researcher contact and supervision. The school division was interested in determining how their primary students would benefit from the ABRA program while introducing a semester long professional development component to their kindergarten and grade 1 teacher in tandem. This school division had previously established a relationship with the lead researcher (Piquette, 2012) and wanted to continue pursuing free access materials that would benefit the students, parents, and teachers in their district. Systematic training was set out for the school calendar team, beginning with a 2 days professional development focus on generic early language and literacy acquisition and strategies, led by the district Early Learning team, followed by a second day of ABRA training led by the research team, Drs Piquette and Savage. All Kindergarten and grade 1 teacher in the school district were involved in this initial 2-days workshop. Specific training continued with the teachers throughout the remainder of the school term, four led by the school district teams and one additional ABRA workshop led by the lead researcher. The school district team was in continual contact with the research team in regards to the ABRA implementation and related classroom based activities. It is important to note that the teachers and administrators agreed and complied with the decision that the professional development activities around ABRA would be used in the experimental classrooms during the ABRA intervention whereas the control classrooms would introduce the ABRA program only after the research phase was conducted. Thus, as the goal of ABRA is to build the literacy skills of young students through trained facilitators, this notion of training the teachers to run the ABRA program and supervising/consulting from a distance provided an opportunity to further evaluate the external validity (effectiveness) of ABRA when run, as it was designed to be, by regular school staff, as a classroom cluster RCT study.

The main aim of this research study was to effectively train and support the regular classroom teachers in their execution of a 10-weeks, whole class ABRA program in order to enhance their student’s early literacy skills. The main question for this research study was to determine whether ABRA yields significant advantages for intervention over control classrooms in early literacy at post-test, using classroom as the unit of analysis.

## MATERIALS AND METHODS

### DESIGN

This study is a cluster RCT intervention study that took place over one academic year in 2008–2009. This study used a pre- post-test experimental intervention design and randomized 24 participating classrooms containing (*n* = 107 kindergarten and 96 grade 1 children in 24 classes, *n*= 203 students) in a rural northern school district in Alberta, Canada. Randomization took place within schools at the classroom level. Pairs of classrooms were identified within each school and were then randomly allocated to either ABRA intervention (*n* = 12 classrooms) or control non-ABRA regular classroom teaching conditions (*n* = 12 classrooms) to reduce bias in cluster RCT designs (see Puffer et al., 2005). Researchers drew names containing the teacher name and grade from each participating school to achieve randomization. This study involved all elementary schools in a participating school district; hence, school board administrators and pragmatic constraints determined the participant sample size. It was anticipated, however, that with *n* = 24 classrooms that the study had modest power to detect medium-to-large effect sizes for intervention.

In order to ensure that the intervention component could be delineated in this study, the importance of teachers continuing with their “regular” educational routines, with the exception of the use of ABRA, was explained to all teachers prior to the study and revisited during the ABRA training sessions as well as during the scheduled classroom observation sessions. Therefore, students in the control classrooms continued to receive their regular instruction and delivery of their English Language Arts (ELA) lessons without an introduction to the ABRA program, while those in the experimental group had ABRA integrated into their ELA lessons. For the experimental classes, ABRA was infused into regular classroom teaching rather than provided as a supplemental program. Intervention teachers implemented the ABRA web-based literacy program 2 h per week. The literacy lesson time remained the same for the control and experimental groups as the experimental group teachers included the ABRA lessons and activities without alteration to the provincially mandated time allocated to language arts.

The teacher participants all attended two full days of professional development during which, on the second day the research study was introduced and a brief overview of ABRA was presented, as well as establishing compliance with participant consent (e.g., parents and their children, school, administration, and teachers). Subsequent training and ongoing support for the participant teachers was also provided by the research team and school board personnel, consisting of four half day sessions focused on language and literacy enhancement, strategies for infusing ABRA activities, and the sharing of teacher based classroom activities to promote early language and literacy concepts All of the teachers in the school district were invited to attend these sessions with the explicit understanding that only the teachers in the experimental control group could use ABRA for the initial 10 weeks cycle. There was one additional full day provided for all teacher participants for the ABRA research at the 5-weeks intervention time period, in which the lead researcher focused on extension activities from the ABRA program. Pretesting occurred prior to the introduction of the ABRA program. In the experimental group, the interventions lasted 2 h per week, typically broken into two sessions of 1 h duration during the week. Classroom observations were conducted by senior board staff who were trained and monitored by the lead researcher, these observations were conducted only a weekly basis for an hour long period, and post-testing took place immediately after 10 weeks of intervention.

### SAMPLE AND PARTICIPANT SELECTION

#### Classroom teacher recruitment

The researchers decided in late spring of 2008 to conduct research on ABRA as well as to provide professional development training to the school district’s primary teachers. Prior to the start of the 2008–2009 school year during a district wide PD day, the researchers provided an overview of the importance of early language and literacy, the ABRA program ^4^, and the research study components (e.g., pre- and post-tests, time commitment, and classroom observations) for all of the teachers in kindergarten and grade 1. The Superintendent, Director of Early Learning and the Director of Technology of this school board were involved in all of the professional development activities and continued to work closely with the researchers throughout the entire study.

The Director of Early Learning sent out an internal email to all kindergarten and grade 1 teacher that contained an expectation to attend a 2-days professional development workshop on early language and literacy with an introduction to the ABRA program. Within this email there was also a request and strong encouragement to consider participation in the research. Teacher consent was sought (primary researcher’s University Human Subjects Ethics Review Board) and attained prior to classroom work, as was the parental and children consent. Teachers were not required to take part in the study, nevertheless, there was a 90% consent rate from kindergarten and grade 1 teacher to take part in the study across the school board. A researcher-created document further outlined expectations for involvement, the nature of professional development, and gave information pertinent for informed consent. Teachers who were interested in participating in the study contacted the Director and were supplied with the date for the next meeting with the researcher team. Consent forms were completed and consent information revisited during the next training session in order to ensure complete understanding of participation and randomization procedures. Randomization was undertaken within schools at the classroom level. Beyond teacher participation consent, the Principals of participating schools were informed of the random allocation of classrooms to the intervention or control condition, to ensure that the experimental classes would be guaranteed 2 h of computer access each week for ABRA implementation, and to ensure that the teachers assigned to the experimental condition could attend ABRA training sessions. The Principals of the participating schools were also ensured that the control classroom teachers would receive additional ABRA training and support after the intervention was completed. Thus all participating schools received free ABRA teacher technological and pedagogical training and support.

Due to the geographic location of this school district, all participating schools were rural. In total, kindergarten and grade 1 teacher from 28 classrooms spread across nine schools initially consented to participate in this study. An additional four teachers from the French Immersion stream requesting to participate in the ABRA training sessions for their own professional development purposes only, but did not feature in the analyses presented here. Due to extenuating circumstances, four of the 28 teachers had to withdraw their participation during the research study (e.g., illness, pregnancy, etc.) prior to randomization. The final ABRA RCT dataset contained 24 classrooms (*K* = 12; grade 1 = 12) paired (six pairs at each grade level), with a total of 203 students (*K* = 107; grade 1 = 96).

#### Student participants

After receiving classroom teacher consent to participate in the ABRA study, the researchers sought permission for students to participate. The research study information and request for consent were provided to the teachers who sent them to the parents of all students in these classrooms. It was clearly stated in the information package that all students would have access to the ABRA program (experimental classrooms initially and after the research was complete, the control classrooms would be supported with the ABRA program) with the student information shared with the researchers only if parental consent was provided. In hindsight, the parents received the request for participation and consent forms without a meeting with the researchers to fully explain the research process; hence, the number of parents who decided to participate in the research component was relatively modest, with a final consent of 203 students to participate. This child sample included 107 kindergarten students (*n* = 48 ABRA, *n*= 59 Control), and 96 first graders (*n*= 57 ABRA, *n*= 39 Control). By gender, the final sample of children consisted of 94 girls and 108 boys. No student was excluded due to language or exceptionalities.

### PROCEDURE

#### ABRACADABRA sessions

An initial 2 days professional development training, which was aimed at providing a foundation for early language and literacy acquisition, followed by the second day with two of the three researchers leading ABRA training was seen as an essential foundation for the teacher’s growing knowledge of early literacy, technology supports, and the ABRA program implementation. All kindergarten and grade 1 teacher were invited to this professional development activity, followed by four half-day workshops led by the school district with consultation and direction provided by the first author. These workshops focused on essential components for early literacy, for example, oral language, phonological awareness, phonemic awareness, word identification, fluency, vocabulary development, and comprehension. The early literacy concepts were explicitly connected with the ABRA program for the intervention teachers during these workshops.

During the full day training, teachers were exposed to the philosophical, developmental, and pedagogical underpinnings of the software and were given hands-on time to explore the software. A theoretically based developmental progression through ABRA activities was emphasized throughout training. For example, teachers were shown how the ABRA phonic activities follow theoretically prescribed patterns of expected difficulty [e.g., detection tasks before production tasks, two-phoneme blends (e.g., ‘a’-‘t’) progressing up sequentially to six-phoneme blending tasks (e.g., ‘s’-‘p’-‘r’-‘i’-‘n’-‘t’), and the early emergence of boundary consonants over medial vowels in word recognition and phonological tasks, the asymmetric later introduction of segmenting tasks, introduction of singleton letter-sounds before complex digraphs, etc.]. As the teachers became more familiar with ABRA during the session, they were encouraged to interact with the various online activities and plan for student progression. Teachers were made aware that ABRA is only a tool, and not an ICT ‘magic bullet.’ Specifically, they were shown through the extended training that it requires highly skilled teachers to implement it well and to link it effectively to cross-curricular learning outside of ABRA sessions.

Once the intervention teachers had some hands-on exposure to the program, the investigators then presented and reviewed a suggested format for the teachers to use during a 1 h ABRA lesson that specified 10 min of ‘word-level’ work, 10 min of ‘text-level’ work, 20 min of collaborative work and 20 min of extension activities. The word-level work involved activities such as letter knowledge, phonological awareness, phonics, and word building. Text-level work invited use of the fluency and comprehension activities based on the Digital Stories component in ABRA. For reading fluency, activities such as high frequency words, reading with expression, reading accurately, and choral reading were suggested. For comprehension, activities that focused on prediction, comprehension monitoring, story elements, and summarizing as well as vocabulary and writing were identified. Emphasis was thus placed on demonstrating that ABRA acts as a ‘balanced’ literacy program (e.g., Pressley, 1998).

All intervention teachers were encouraged to select activities relevant to the appropriate point in children’s development. Thus, for kindergarten teachers, the phonic activities might be more likely to start with simpler activities within ABRA such as sound awareness, syllable and word counting and aspects of rhyme awareness, whereas the teachers in grade 1 might move their children more quickly to blending tasks. Similarly, for comprehension and aspects of the fluency tasks, teachers of kindergarten children were asked to encourage children to listen to stories and then complete tasks such as story ordering and summarizing, whereas teachers of grade 1 children were asked to encourage children to read the texts and then complete comprehension tasks. This differentiated use of ABRA was also encouraged through the sustained in-class follow-up support for teachers by the school district personnel. These personnel members continued to support their teachers and remained in contact with the lead researcher throughout the school term. Furthermore, as stated earlier, the school district ran four additional professional development sessions based on the early language and literacy skills addressed in our ABRA sessions.

A second ABRA workshop for the intervention teachers only was set at the 5-weeks mark during the intervention research cycle in order for the lead researcher to provide additional support on ABRA, share strategies regarding how to use ABRA resources, and to invite the participants to collaborate on future ABRA based lessons. During this ABRA workshop the conversations between the lead researcher and the intervention teachers evidenced how they were using the program in innovative cross-curricular formats.

*Collaborative work* encouraged students to work together in order to practice and strengthen skills they learned in the earlier two sections. Collaborative work did not have to be conducted on a computer. For example, we suggested that students could write alternative endings for the digital stories with a peer, engage in readers’ theater, put on a puppet show, and so forth.

*Extension activities* often included additional opportunities for the students to engage in collaborative work. For example, after reading *The Fruit Family* story, a kindergarten teacher could have her students draw pictures of the different types of fruit that they ate and label their pictures.

Teachers were informed that while regular access to appropriately leveled and progressively more demanding word-level and text-level activities were required, this suggested curriculum was a fairly flexible guideline and should be adapted to meet the individual needs of their students as well as their own teaching styles. Teachers also had freedom to run the intervention as whole class, small group, individual, or some combination of the groupings. It was known at training that teaching would also vary depending upon access to technology in particular schools (e.g., presence of SMART boards or multiple computers in classrooms, use of a distinct ICT room in school). The responsibility for developing appropriate specific lesson plans and interventions always rested with the regular classroom teachers.

All of the teachers received a hard copy of an extensive ABRA *“Teacher’s Manual”* that illustrated in detail how ABRA could be used in these domains. Teachers also visited the “*Teacher’s Zone*” available online as ^5^, a resource area for them. Finally, the teachers got into small groups based on the grade levels they taught, and planned ABRA introductory lessons for their classes based on the suggested 1-h format.

*Classroom computers* are seen as a necessary learning tool in Alberta, hence, it is quite common for every classroom to have 2–3 computers for student use, an electronic interactive whiteboard for collaborative learning, and a dedicated computer laboratory for whole classroom use. The rural school district excelled in technology with multiple computers in each classroom, interactive whiteboards in each classroom, a computer lab and additional computer carts with 20–25 computers that could be wheeled into a classroom when needed. In addition, much time was spent on professional development activities for all staff in this school district to ensure that they were comfortable using and teaching with computers and interactive whiteboards. Each experimental classroom had access to 30 computers during the experimental phase of the study. The student–computer ratio was 1 to 1 and all students had access to the teacher led interactive whiteboard that projected the ABRA activities as a focal point within instruction, practice, and discussion. Computer assistance was provided for the students through their trained grade level teacher and a teacher’s assistant. As ABRA was embedded into the regular classroom teaching, the experimental group accessed the ABRA tool in their own classroom for the majority of the time periods. Technology support was provided within each school by the computer instructor and overseen by the school district’s Director of Technology who was an integral ABRA research team member. There were no technical issues or concerns with the computer technology, interactive whiteboard use, computer applications or accessibility of the computers for the students.

#### Project management and roles

The Director of Early Learning for this school district requested they take up the position as lead coordinator for this study as it would provide experience necessary for a graduate degree that was underway. This director coordinated the execution of the study under the supervision and support of the first author. The coordinator assisted with the recruitment and overseeing the training of RAs for pre and post-testing as well as to conduct classroom observations. The RAs were predominantly school district teachers who were currently taking a graduate level degree and had established themselves as lead teachers who desired further successful applied development within the language and literacy domain (Piquette-Tomei et al., 2009). The RAs as well as the coordinator visited the 24 participating classrooms and administered the pre-and post-test measures. The researchers and coordinator were available to provide technical support and answer general pedagogical questions regarding the utilization of ABRA throughout the intervention phase. Weekly telephone meetings were held between the first author and the study coordinator to update and address questions about the ABRA program, data collection and literacy lesson support.

***Literacy Assessment Measures***. The ABRACADABRA research study intervention was designed to aid alphabetics, phonological awareness, word reading, and comprehension, hence, reliable and valid psychometric tests were selected to examine all of these component abilities.

#### Letter-sound knowledge

To assess letter-sound knowledge, participant were shown the 26 letters of the English alphabet and asked the student to say the corresponding sound of each letter presented following the assessment and scoring system described by Savage et al. (2009). The test yields a raw score with a maximum of 26. The Spearman–Brown split-half internal reliability of this test in nationally representative Canadian samples (Savage et al., 2013) at pre-test is *r*= 0.87.

#### Blending words

This measure assesses a child’s phonological blending ability. A subtest of the *Comprehensive Test of Phonological Processing* (CTOPP) was used to examine students’ ability to blend words (Wagner et al., 1999). In this test, the children listened to a series of disjointed sounds and then blend the sounds together to make a whole word. The test yields an age-equivalent standard score. The Spearman–Brown split-half reliability coefficient for this measure in nationally representative Canadian samples (Savage et al., 2013) at pre-test is *r*= 0.86.

#### Fry words

To assess the students word reading skills, a test was adapted using words from the *Fry’s Instant Word List* (Fry et al., 2000). Twenty words were randomly selected from Fry’s first 200 words. The same 20 words were used at pre- and post-test. Each of the selected 20 words were placed on individual index cards and shown one at a time to participants. The students read each word presented to them, and received a point for each word correctly read, yielding a raw score, with a maximum for this test of 20. The Spearman–Brown split-half reliability of this test in the present sample in nationally representative Canadian samples (Savage et al., 2013) at pre-test is *r*= 0.89.

#### The Group Reading Assessment and Diagnostic Evaluation (GRADE)

The Group Reading Assessment and Diagnostic Evaluation (GRADE) is a standardized, nationally normed, instrument designed to be administered to either the whole class or individually (Williams, 2001). The GRADE is reported to have strong internal consistency (*r* = 0.95–0.99) and retest reliability (*r* = 0.80; Williams, 2001). Reviews of GRADE (Fugate, 2003; Waterman, 2003; McBride et al., 2010) have concluded that this tool is a reliable and valid measure of early reading ability.

The GRADE *Word Reading test* was used to assess word-reading skills. For the word recognition test, participants were asked to identify the word read by the examiner from a choice of four visually and/or phonologically similar words. The test consisted of 20 words sets. The standardized assessment yields a stanine score for this subtest alone. The published Spearman–Brown split-half internal reliability coefficient for this measure in nationally representative U.S. samples is *r*= 0.80 (kindergarten) and *r*= 0.90 (grade 1).

The *Listening Comprehension* subtest of the GRADE was used to assess the students’ understanding of spoken language. Children are read sentences and then asked to select a picture from four choices that best illustrates the meaning of each sentence. The standardized assessment yields a stanine score for this subtest alone. The Spearman–Brown split-half internal reliability coefficient for this measure in nationally representative Canadian samples (Savage et al., 2013) at pre-test is *r*= 0.89.

### TESTING PROCEDURE

All participants completed the letter-sound knowledge, Fry words, blending words, and GRADE listening comprehension and word reading measures. All children were seen twice at both pre- and post-test for testing. The first session involved individual testing of children. Here the Fry words, letter-sound knowledge, and CTOPP blending tasks were administered. The second session used a whole-class group testing approach of all GRADE measures with the classroom teacher assisting the RA with the test administration to ease any potential student discomfort with testing.

### TREATMENT FIDELITY

In this study, treatment fidelity was validated through observations and recording, and teacher questionnaires. The study coordinator and a lead RA were tasked with independently visiting a classroom while utilizing a rubric which focused on the implementation of ABRA in the classrooms and to record observations from the classroom. These rubrics and observations were compared and discussed, their findings that there was close alignment in agreement regarding the observed classrooms. It was found that the teachers, who were informed during their training that adequate treatment integrity would consist of 20 h of exposure to ABRA for each student in the intervention condition with adequately implemented ABRA lessons (e.g., evidencing careful planning, differentiation, and progression) did indeed meet this criteria. The observations also confirmed there was no ABRA teaching in the control condition classrooms.

To obtain further information about the quality of teaching for both the experimental and control classroom, the coordinator and lead RA conducted observations using the Early Literacy and Language Classroom Observation (ELLCO, Smith et al., 2002). The ELLCO is a standardized instrument assesses both the global quality of the classroom (e.g., classroom climate, approach to management, classroom organization), as well as language, literacy, and curriculum. The coordinator reported that each of the classrooms reflected good to above average ratings regarding the quality of classroom supports for literacy.

## RESULTS

### PRELIMINARY DATA ANALYSES

Preliminary data analyses of child- and classroom-level data spreadsheets using conventional levels for alpha, suggested that there was no marked kurtosis or skew in the classroom-level attainment data. All classroom-level variables were within acceptable limits of normality for skewness and kurtosis, so no data transformations were undertaken. There was no strong evidence of outliers. At this point, no data was excluded from analysis.

#### Student-level missing data

The pattern of student-level missing data was inspected using the SPSS MVA package to consider the randomness and impact of missing data and to then most-appropriately impute missing values where appropriate. Missing data represented less than 5% of the total data across all variables and so most common procedures for dealing with missing values would yield similar results (Tabachnick and Fidell, 2007). Analyses were run using conventional χ^2^ to contrast the proportion of missing versus present data in intervention versus control group conditions at pre- and post-tests. There was no evidence of selective experimental mortality across conditions: χ^2^(1) < 1, n.s., in all cases. Further analysis using MVA found full-sample child-level data were also ‘missing completely at random’ for all variables, using Little’s MCAR test, (*p* > 0.05 in all cases). Regression-based imputation procedures were selected, with reading pretest variables serving as predictors. The mean of the fifth iteration were selected for analyses.

### MAIN DATA ANALYSES

The classroom-level mean and standard deviation of all classroom-level attainment variables at pre- and post-test are presented in **Table 1**. Inspection of **Table 1** shows signs of post-test advantage for the ABRA group on letter-sound knowledge, phonological blending, and Fry words despite often starting at a lower pre-test level of attainment than controls. Few clear signs of advantage were evident in the grade and listening comprehension measures.

**Table 1:** Classroom-level variable mean, standard deviation and effect sizes in intervention and control groups.

	Intervention group					Control group					
	Pre-test		Post-test			Pre-test		Post-test			
Measure	*M*	*SD*	*M*	*SD*	Cohen’s *d*	*M*	*SD*	*M*	*SD*	Cohen’s *d*	Value Added *d*
LSK	10.67	7.32	18.62	4.18	+1.33	11.79	8.71	16.96	6.43	+0.68	+0.65
Fry words	1.95	2.61	5.02	5.24	+0.74	2.68	3.45	5.50	5.85	+1.14	–0.40
Blending words	6.43	0.63	7.82	2.50	+0.76	6.90	2.53	7.48	2.38	+0.24	+0.52
Word reading	2.64	1.91	2.27	1.01	–0.24	3.75	1.66	2.75	0.87	–0.76	+0.52
Listening comprehension	3.36	0.67	4.36	0.81	+1.34	3.50	0.80	5.00	1.41	+1.31	+0.02

*LSK, letter-sound knowledge; Fry words, 20 randomly selected Fry words from most frequent words; blending words, CTOPP blending words subtest age equivalent scores; word reading, GRADE word reading subtest stanine score; Listening comprehension, GRADE listening comprehension subtest stanine score.*

### DOES A CLASSROOM-LEVEL CLUSTER RCT EFFECTIVENESS TRIAL OF ABRA YIELD SIGNIFICANT ADVANTAGES FOR INTERVENTION OVER CONTROL CLASSROOMS IN EARLY LITERACY AT POST-TEST?

In this cluster RCT, random allocation of students took place at the classroom-level, producing a nested design in which likely contextual influence classrooms on the achievement of the individual participants can be evaluated (e.g., Raudenbush and Bryk, 2002; Hox, 2010). Our data were thus first analyzed with HLM with randomized classroom as the unit of analysis. The final HLM models were built in standard ‘bottom-up’ fashion from preliminary analyses with steps in HLM followed sequentially in order to yield the final models. Model 1 was an Unconditional One-way ANOVA Model with Random Effects and confirmed that there was classroom level variance at pretest and post-test on attainment measures beyond variance attributable to pupils, that HLM was appropriate.

Subsequent hierarchical ANCOVA models tested whether candidate covariates are significant and should be retained in the final model. A hierarchical ANCOVA model was appropriate in this design as pretest attainment and chronological age was always a significant covariate of its corresponding post-test measure. The final three-level hierarchical model examined, built on these tested assumptions above, sought to establish whether the significant classroom-level variance on post-test attainment measures (after control for school-level shared variance at level 3, pretest classroom-level attainment variance at level 2, and pre- and post-test pupil level attainment variance and pupil chronological age at level 1) was explained by the ELA with ABRA versus ELA without ABRA factor. Equations 1, 2, and 3 describe this final model at the pupil, classroom, and school levels, or student i in classroom j in school k, respectively.

(1) Equation for Student Level 1 Model:

$$Y_{ijk}\,\;=\pi_{00k}+\pi_{1jk}(PRETESTi_{jk})+e_{ijk}$$

(2) Equations for Classroom Level 2 Model:

$$\pi_{00k}\,\;=\beta_{00k}+\beta_{01k}*(PRETEST\,ATTAINMENT_{jk})+\beta_{02k}*(INTERVENTION_{jk})+r_{0jk}$$

(3) Equations for School Level 3 Model:

$$\beta_{00k}=\gamma_{000}+\mu_{00k}$$

In these analyses, predictor variables were left uncentered and ratio-level raw scores which have a meaningful zero point value were used so as to ease interpretation. Equations 1–3 also show that the slope coefficients for all independent variables are treated as fixed: they are not allowed to randomly vary across classrooms and schools.

The results of these analyses are reported in **Table 2**. This analysis is of five measures: letter-sound knowledge, Fry words, phonological blending, and GRADE word reading and listening comprehension across all *n* = 24 paired classrooms. These results show that there is a significant effect of ABRA on intervention mean = vs. control group mean contrasts for letter-sound knowledge (*t* =2.56, *p* < 0.01). No other effects, however, reached significance (*t* < 1.8 in all cases).

**Table 2:** Hierarchical linear model results for the effect of ABRA condition on post-test attainment.

Variable	Classroom-level Model	
	Coefficient	*SE*
**Letter-sound knowledge 2 = dependent variable**
Letter-sound knowledge 1	0.11	0.49*
Intervention condition	3.04	1.19**
**Fry words 2 **=** dependent variable**
Fry words 1	2.11	0.83**
Intervention condition	0.11	1.00
**CTOPP phonological blending 2 = dependent variable**
CTOPP phonological blending 1	0.13	0.03***
Intervention condition	0.14	0.39
**GRADE word reading 2 = dependent variable**
GRADE word reading 1	0.23	0.11*
Intervention condition	0.21	0.20
**GRADE listening comprehension 2 = dependent variable**
GRADE listening comprehension 1	0.11	0.05*
Intervention condition	0.29	0.26

*p < 0.05, **p < 0.01, ***p < 0.001.

*Suffix numerals 1, pretest; 2, post-test*.

In addition to analyses of main statistical effects effect sizes are reported for interventions in **Table 1**. Following Cohen (1988), standard effect sizes (*d*) were first calculated. These were pre-post-test differences over the pooled sample standard deviation. Beyond this, and arguably most informatively in interpreting the specific effects of intervention over the effects of control (non-ABRA) teaching, *value-added* effect sizes are reported: these are the difference in standard effect sizes (intervention *d* minus control *d)* and could be understood to reflect the gain in attainment more specifically attributable to the ABRA intervention than to general teaching *per se*. Inspection of these latter data in **Table 1** showed medium positive *value-added* effect sizes were evident for three of five outcome measures: letter-sound knowledge (*d*= +0.66), phonological blending (*d* = +0.52), and word reading (*d* = +0.52 for the GRADE word reading measure but *d* = –0.40 for the Fry words measure), for the intervention condition over effect sizes for control group regular teaching.

## DISCUSSION

The main aim of the present study was to undertake a well-designed cluster RCT trial to evaluate the added value of ABRA delivered by regular classroom teachers to all children in their regular K-grade 1 ELA classes, to replicate and extend the findings of the only other published field-based external validity (effectiveness) trial of ABRA to date (Savage et al., 2013). The results indicated that ABRA produced statistically significant effects on measures of letter-sound knowledge. This specific pattern of effects of ABRA on letter knowledge replicates patterns reported in four previous researcher-led internal validity trial studies of ABRA (Comaskey et al., 2009; Savage et al., 2009; Wolgemuth et al., 2011, 2013). The results of the present study also replicate the findings reported in the only existing external validity cluster RCT trial of ABRA (Savage et al., 2013). Like the Savage et al. (2013) study, this study was a field RCT run by regular class teachers for unselected samples of children in regular classrooms where ABRA, linked to regular whole class teaching was contrasted with regular teaching without ABRA. The present study thus represents an important novel contribution to knowledge as this is the first replication of the results of an ABRA *effectiveness* study, that is, a study run by trained regular classroom teachers rather than an *efficacy* study run by graduate research students from universities (Comaskey et al., 2009; Savage et al., 2009) or trained educators employed specifically to run the intervention (Wolgemuth et al., 2011, 2013), and cautiously suggest that trained teachers using ABRA can cause greater improvements in reading even in relatively remote rural school board settings in Canada. In addition the fact that the sample of teachers represented 90% of all k and grade 1 teacher in this school board, suggests that the ABRA model is both accepted by, and can be readily implemented by, a majority of teachers, in this one context at least. Such acceptance or ‘efficiency validity’ is often seen as crucial to wider scale-up implementation of practice-relevant RCT trial findings (Dunst and Trivette, 2012). Furthermore, the unique flexibility within the ABRA implementation and intervention should be noted. It is important to recognize how the participating teachers appropriated the intervention in their respective classrooms, how the training emphasized the adjustment of individual components for specific contextual use, and how the school district and researcher support with the teachers all converged to increase teacher engagement, commitment and success with ABRA.

Contrary to hypotheses, there were no significant overall effects of the ABRA intervention condition on measures of listening comprehension, phonological blending, and word reading in inferential analyses. However, analyses of effect sizes showed medium value-added effect sizes for intervention on 3 of 5 measures with only listening comprehension and Fry words showing no positive effects in either form of analyses. lt should be noted that the classroom-level sample size which forms the basis of analyses (*n*= 24 classrooms, *n* = 203 children) was relatively modest in size for HLM analyses, so it was anticipated that quite strong effects might be evident in effect size analyses but not always in inferential analyses. Theoretically, the development of letter knowledge and phonological awareness is closely allied to the ability to ‘decode’ novel words. Letter-sound knowledge, phonological blending skills and early reading have for these reasons, appropriately been called ‘foundations of literacy’ (e.g., Seymour, 1997; Byrne, 1998).

The absence of effects for ABRA on listening comprehension is inconsistent with an internal validity trial reported by Savage et al. (2009) and the recent results in Kenya reported by Abrami et al. (2014), but consistent with the only previous external validity trial of ABRA (Savage et al., 2013). Savage et al. (2013) speculated that there are a number of possible explanations for these latter patterns. First, there is relatively limited evidence that teachers in the North American context use explicit strategy teaching for comprehension even outside of ICT (e.g., Pressley, 1998), and little teaching of comprehension with ABRA was noted. Secondly, the ABRA intervention was run toward the beginning of the academic year with many beginner readers. It is possible that teachers feel more comfortable working on comprehension later in the year (Deault, 2011; Deault and Savage, 2013).

In sum, the present research joins a recent and growing literature of high quality RCT studies from around the world, e.g., in France (Ecalle et al., 2009), the United States (Chambers et al., 2008), Australia (with ABRA, Wolgemuth et al., 2011, 2013), Finland (Saine et al., 2011), and Canada (with ABRA, Comaskey et al., 2009; Savage et al., 2009; Di Stasio et al., 2012; Abrami et al., 2014) showing that bespoke literacy technology, when used in regular classrooms by thoroughly trained teachers, and linked through their high-quality classroom teaching, as part of language arts, can impact early literacy.

### LIMITATIONS OF THE PRESENT STUDY

A number of potentially important potential limitations in the present study need to be noted. First, the modest sampling and sample size limits generalization of findings undertaken as this study was in one school board. This research nevertheless sits alongside other studies using ABRA, and most notably that of Savage et al. (2013) that used a relatively large sample of 74 classrooms in three provinces of Canada, that reported similar findings. We would also argue that while the study was relatively modest in scale, if a teaching approach or tool is to be *practically* useful it would need to show up in such school-board level data as we present here. Our effect sizes in particular do suggest that while the study is modest in scale, practically useful effects were nevertheless evident.

The modest sample size also appeared to be directly related to parental knowledge that all of their children would be receiving the benefits of the ABRA instruction in the classroom, regardless of consent for the secondary use of data for publication. The hypothesis was that this resulted in a relatively low rate of student consent, as there was no “pressing need” for research participation. The current results did yield comparable numbers of children in the control and intervention conditions of the study, though there were consistent differences between the intervention and control groups at pre-test (generally favoring controls). In the consenting sample at least (*n* = 203), there was no evidence of selective mortality. Nevertheless, in the future while we would not alter the full access to ABRA we are mindful that additional information for the parents regarding the research cycle and benefits of research itself would be necessary. This type of information could be provided by a parent information evening or through an appendix to the request for participation and consent form.

The results are also potentially limited by reporting effects at immediate post-test and not also at delayed post-test. However, a pre-condition for obtaining the capacity to randomize interventions in schools was our promise to train the control class teachers after the intervention period was completed. Much longitudinal research including our own work with ABRA strongly suggests that phonological and letter-sound skills that improved here are foundational for later reading comprehension. Longitudinal data from our other ABRA studies suggests this pattern does indeed obtain (Di Stasio et al., 2012). In the present study, we cannot completely rule out the possibility that results reflect a more general motivational impact of awareness that a study is being run rather than ABRA and teaching per se (e.g., ‘Hawthorne’ or ‘John Henry’ effects). However, while more evidence is needed on the issue, the findings from post-test follow-up data alongside other evidence from treatment integrity analyses that link gains in reading to the directly observed quality and consistency of program implementation for ABRA (Savage et al., 2013; Wolgemuth et al., 2014) are less consistent with a purely motivational interpretations of findings.

### IMPLICATIONS AND FUTURE STUDIES

It has been argued that the single biggest challenge facing reading researchers, implementation scientists, and practitioners in the 21st century is the issue of building scalable and sustainable interventions (Abrami et al., 2008). We demonstrate here through replication of a cluster RCT intervention design, the robustness of the impact of teaching that incorporates ABRA on young children’s literacy. As a replication of a field-based trial these results suggest that ABRA an open, free-access resource can be used effectively at scale in school boards even in relatively remote contexts. ABRA has also been used efficaciously in remote regions of northern Australia (Wolgemuth et al., 2011, 2013) and Kenya (Abrami et al., 2014), so the model of teaching with ABRA thus has potential to be used by communities worldwide to aid literacy. Some years ago Miller (1969) argued that the best and most useful findings of scientific psychology should be ‘given away’ to the community. We thus view ABRA as a community resource for teaching in this long-established spirit of this ‘giving away’ of psychological findings (Savage et al., 2013). The present findings add to the confidence that this can in fact be done in ways that measurably improve early literacy for whole classes of children, and which teachers may find acceptable. One of the next steps therefore must be to explore both the scale-up of RCT interventions nationally and internationally as part of explicit evidence-driven national policy initiatives. In addition, now there is accumulating evidence both for the effectiveness and efficacy of ABRA-supported teaching from RCT studies, it will be important to gain further insights, using a range of methodologies on the richer picture of *how* teachers use ABRA effectively in their regular teaching, and the support teachers and other school professionals need to encourage both high-level adaptations and strong expectancies of success, and how to engender community engagement in technology-based and other forms of literacy for effective intervention. In addition, the long-term effects of intervention, ways to use evidence to design better and more effective ABRA activities, *how* ABRA-linked teaching improves literacy process, and how to best hand-over ABRA effectively to school boards remain highly productive research avenues. All of this work is the next goal of future studies.

## Conflict of Interest Statement

The authors declare that the research was conducted in the absence of any commercial or financial relationships that could be construed as a potential conflict of interest.

## Footnotes

1 http://petitabra.concordia.ca

2 http://abralite.concordia.ca

3 http://grover.concordia.ca/abracadabra/

4 While student stories from Australia and Kenya have been added to ABRA, the key components of the tool described here remain essentially unchanged from that used by the teachers in this study.

5 http://abralite.concordia.ca/pd/index.php
